# Supplementary material for: Birth weight is associated with brain tissue volumes seven decades later but not with MRI markers of brain ageing
Source: Neuroimage Clin. 2021 Aug 3;31:102776. doi: 10.1016/j.nicl.2021.102776 (PMC8358699; doi:10.1016/j.nicl.2021.102776)
Supplement: Supplementary data 1 [file mmc1.docx]

Supplementary Information for

Birth weight is associated with brain tissue volumes seven decades later, but not with MRI markers of brain ageing

Emily Wheater^1^, Susie Shenkin^2,4^, Susana Muñoz Maniega^3,4^, Maria Valdés Hernández^3,4^, Joanna M. Wardlaw^3,4^, Ian J. Deary^4,5,6^, Mark E. Bastin^3,4,6^, James P. Boardman^1,3^, Simon R. Cox^4,5,6^.

* Simon R. Cox

**Email:**  [simon.cox@ed.ac.uk](mailto:simon.cox@ed.ac.uk)

**This PDF file includes:**

Tables S1 to S5

Figure S1

Table S1. Tract loadings on general factor of fractional anisotropy

| Tract | Standardised loadings. |
| --- | --- |
| Splenium | 0.425 |
| Genu | 0.624 |
| LArc | 0.613 |
| RArc | 0.606 |
| LATR | 0.600 |
| RATR | 0.585 |
| LCing | 0.520 |
| RCing | 0.530 |
| LUnc | 0.634 |
| RUnc | 0.661 |
| LILF | 0.437 |
| RILF | 0.416 |

*Note.* the genu and splenium of the corpus callosum, LArc – Left arcuate, RArc – right arcuate, LATR – left anterior thalamic radiation, RATR – right anterior thalamic radiation, LCing – Left cingulum bundle, RCing – Right cingulum bundle, LUnc – left uncinate, RUnc – right uncinate, LILF – left inferior longitudinal fasciculus, RILF – right inferior longitudinal fasciculus.

Table S2. Associations between birth weight and the ratio of global brain volumetric MRI measures as a proportion of intracranial volume, correcting for age and sex

|  |  | |
| --- | --- | --- |
|  | *β* | *p* |
| TB/icv | 0.006 | 0.937 |
| GM/icv | -0.074 | 0.379 |
| NAWM/icv | 0.149 | 0.080 |
| WMH/icv | -0.094 | 0.273 |

*Note.* Standardised regression coefficients between birth weight and volumetric MRI measures expressed as a ratio with ICV. Bold typeface denotes FDR *q* < 0.05. TB: total brain volume; GM: grey matter volume; NAWM: normal appearing white matter volume; WMH: white matter hyperintensity volume.

**Table S3.** Participant characteristics

| Total N | Birth weight and MRI  N = 137 | MRI; no birth weight  N = 521 | Birth weight; no MRI  N = 35 | No birth weight; no MRI  N = 173 |
| --- | --- | --- | --- | --- |
| Female/Male | 63/74 | 243/278 | 16/19 | 96/77 |
| Age 11 IQ | 99.84 (41.98 – 126.27) | 101.14 (38.48 – 129.88) | 97.41 (51.49 – 121.45) | 100.64 (49.44 – 127.61) |
| Mean age (range) / years | 72.6 (71.1 – 74.1) | 72.5 (70.9 – 74.0) | 72.6 (71.8 – 73.3) | 72.6 (70.9 – 73.9) |
| Mean birth weight (range) /g  ≤2500  2501-3000  3001-3500  3501-4000  4001-4500 | 3346 (1843 – 4423)  6  22  58  40  11 | -  -  -  -  -  - | 3221 (2013 – 4082)  3  8  11  11  2 | -  -  -  -  -  - |
| Mean height (range) /cm | 166.0 (146.0 – 185.5) | 166.3 (143.1 – 195.5) | 166.7 (146.5 – 188.0) | 166.9 (143.5 – 191.9) |
| Mean weight (range) / kg | 78.35 (50 – 116.8) | 76.76 (41.2 – 135.0) | 78.9 (47.0 – 102.0) | 78.6 (45.0 – 125.0) |
| Mean BMI (range) | 28.37 (19.3 – 45.7) | 27.71 (16.7 – 47.6) | 28.26 (21.6 – 39.8) | 28.13 (16.9 – 51.0) |
| Smoking (current/ex/never) | 14/55/68 | 40/235/246 | 4/16/15 | 15/72/86 |
| Hypertension (yes/no) | 66/71 | 261/260 | 17/18 | 81/92 |
| Hypercholesterolemia (yes/no) | 62/75 | 214/307 | 16/19 | 64/109 |
| Diabetes diagnosis (yes/no) | 21/116 | 47/474 | 4/31 | 23/150 |
| Cardiovascular disease history (yes/no) | 43/94 | 136/385 | 11/24 | 60/113 |
| Stroke history (yes/no) | 11/126 | 34/487 | 2/33 | 8/165 |

*Note.* Summary of descriptive data for LBC 1936 sample for those with MRI and BW data included in this analysis, those LBC wave 2 participants who had MRI only, those with birth weight and not MRI and those who had neither data type. BMI: body-mass index. Hypertension, hypercholesterolemia, diabetes, cardiovascular and stroke history were obtained by participant self-report in a structured medical interview.

**Table S4.** Associations between birth weight and volumetric MRI measures correcting for age, sex, cardiovascular risk factors and cardiovascular disease history.

|  |  | |
| --- | --- | --- |
|  | *β* | *p* |
| TB | **0.245** | **0.002** |
| GM | **0.182** | **0.019** |
| NAWM | **0.268** | **<0.001** |
| WMH | -0.035 | 0.701 |
| gFA | -0.053 | 0.547 |
| PSMD | -0.016 | 0.850 |

*Note.* Standardised regression coefficients between birth weight and volumetric/white matter microstructure MRI measures. Bold typeface denotes FDR *q* < 0.05. TB: total brain volume; GM: grey matter volume; NAWM: normal appearing white matter volume; WMH: white matter hyperintensity volume; gFA: general factor of fractional anisotropy; PSMD: peak width skeletonised mean diffusivity.

**Table S5.** Associations between birth weight and volumetric MRI measures correcting for age, sex, cardiovascular risk factors and cardiovascular disease and stroke history.

|  |  | |
| --- | --- | --- |
|  | *β* | *p* |
| TB | **0.233** | **0.004** |
| GM | 0.172 | 0.031 |
| NAWM | **0.262** | **0.001** |
| WMH | -0.049 | 0.598 |
| gFA | -0.060 | 0.503 |
| PSMD | 0.010 | 0.910 |

*Note.* Standardised regression coefficients between birth weight and volumetric/white matter microstructure MRI measures. Bold typeface denotes FDR *q* < 0.05. TB: total brain volume; GM: grey matter volume; NAWM: normal appearing white matter volume; WMH: white matter hyperintensity volume; gFA: general factor of fractional anisotropy; PSMD: peak width skeletonised mean diffusivity.


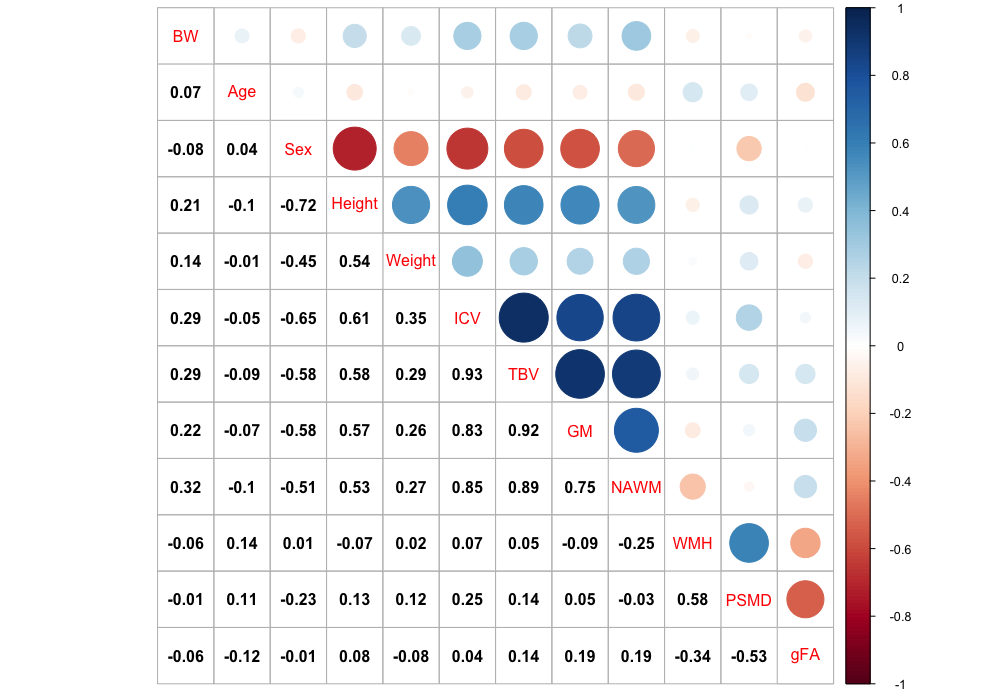


**Figure S1.** Correlation matrix showing Pearson’s r correlations between brain MRI features and birth weight, age at MRI, height and weight, and point biserial correlations between these variables and sex.

Note BW: birth weight; ICV: intracranial volume; TBV: total brain volume; GM: grey matter volume; NAWM: normal appearing white matter; WMH: white matter hyperintensity volume; PSMD: peak width skeletonized mean diffusivity; gFA: general factor of fractional anisotropy.

[Colour]
